# Genome Sequence and Characterization of a Xanthorhodopsin-Containing, Aerobic Anoxygenic Phototrophic *Rhodobacter* Species, Isolated from Mesophilic Conditions at Yellowstone National Park

**DOI:** 10.3390/microorganisms10061169

**Published:** 2022-06-07

**Authors:** John A. Kyndt, Sydney Robertson, Isabella B. Shoffstall, Robert F. Ramaley, Terrance E. Meyer

**Affiliations:** 1 College of Science and Technology, Bellevue University, Bellevue, NE 68005, USA; srobertson011@yahoo.com; 2 Department of Chemistry and Biochemistry, New Mexico State University, Las Cruces, NM 88003, USA; bella4@nmsu.edu; 3 Department of Biochemistry and Molecular Biology, University of Nebraska Medical Center, Omaha, NE 68198, USA; rramaley@unmc.edu; 4 Department of Chemistry and Biochemistry, The University of Arizona, Tucson, AZ 85721, USA; temeyer@email.arizona.edu

**Keywords:** aerobic anoxygenic phototrophs, AAP, *Rhodobacter*, purple nonsulfur bacteria, hot spring, Yellowstone, xanthorhodopsin

## Abstract

The genus *Rhodobacter* consists of purple nonsulfur photosynthetic alphaproteobacteria known for their diverse metabolic capabilities. Here, we report the genome sequence and initial characterization of a novel *Rhodobacter* species, strain M37P, isolated from Mushroom hot spring runoff in Yellowstone National Park at 37 °C. Genome-based analyses and initial growth characteristics helped to define the differentiating characteristics of this species and identified it as an aerobic anoxygenic phototroph (AAP). This is the first AAP identified in the genus *Rhodobacter*. Strain M37P has a pinkish-red pigmentation that is present under aerobic dark conditions but disappears under light incubation. Whole genome-based analysis and average nucleotide identity (ANI) comparison indicate that strain M37P belongs to a specific clade of recently identified species that are genetically and physiologically unique from other representative *Rhodobacter* species. The genome encodes a unique xanthorhodopsin, not found in any other *Rhodobacter* species, which may be responsible for the pinkish-red pigmentation. These analyses indicates that strain M37P is a unique species that is well-adapted to optimized growth in the Yellowstone hot spring runoff, for which we propose the name *Rhodobacter calidifons* sp. nov.

## 1. Introduction

Species of the genus *Rhodobacter* are purple nonsulfur photosynthetic alphaproteobacteria that have unique morphological and physiological properties [1,2]. *Rhodobacter* belongs to the *Alphaproteobacteria* (Pseudomonadota phylum) and representatives are found in a wide range of environments. The two best-studied species in the *Rhodobacter* genus are *Rba. capsulatus* and *Rba. sphaeroides*, which are often used as model systems for bacterial photosynthesis studies. In more recent developments, the use and studies of *Rhodobacter* have been expanded to a wide range of applications, from the consideration of *Rba. sphaeroides* for in vivo imaging of tumor cells with natural advantages over other fluorescent materials [3], to the use of various *Rhodobacter* LPS molecules for TLR-mediated chemokine expression [4,5,6,7], and all the way to the use of *Rhodobacter* extracts as a food supplement to regulate immune responses in humans and animals [7].

Members of the genus *Rhodobacter* all perform anoxygenic photosynthesis. They also fix nitrogen and thereby play key roles in biogeochemical cycles. Most *Rhodobacter* species can grow aerobically, but when doing so repress the synthesis of photosynthetic pigments, and all oxidize sulfide at low concentrations but with differences in the oxidation products. *Rba. blasticus* is an unusual species that produces lamellar membranes and, prior to the recent discovery of *Rba. thermarum*, there were no others sharing this property [8,9]. 

Currently, there are 21 validly named species in the *Rhodobacter* genus: *Rba. aestuarii* [10], *Rba. alkalitolerans* [11], *Rba. amnigenus* [12], *Rba. azollae* [13], *Rba. azotoformans* [14], *Rba. blasticus* [8], *Rba. capsulatus* [1,15], *Rba. flagellatus* [16], *Rba. johrii* [17], *Rba. lacus* [13], *Rba. maris* [18], *Rba. ovatus* [19], *Rba. sediminis* [20], *Rba. sediminicola* [21], *Rba. sphaeroides* [22], *Rba. tardus* [23], *Rba. thermarum* [9], *Rba. veldkampii* [24], *Rba. vinaykumarii* [25], *Rba. viridis* [26], and *Rba. xinxiangensis* [27]. Eight of these were discovered in the last three years and not all of these have their genomes sequenced; however, many metagenome-derived uncultivated *Rhodobacter* genomes are found in NCBI GenBank. More will undoubtedly be added since metagenomic sequencing is becoming more prevalent in environmental analysis. A recent effort to resolve the diversity in the taxonomy of the *Rhodobacter* genus [28] identified the existence of four monophyletic clusters, with interspersing chemotrophic species, and proposed the reclassification of several members of the genus *Rhodobacter* into three new genera: *Luteovulum*, *Phaeovulum*, and *Fuscovulum*. Although a revision of the taxonomy of the *Rhodobacter* was certainly due, that study was performed using the genomic comparison of only 12 strains, and still leaves many of the species designated as *Rhodobacter* dispersed within the larger *Rhodobacter* phylogeny. For the purpose of the current discussion, the *Rhodobacter* designation is used and the new genus names (*Luteovulum*, *Phaeovulum*, and *Fuscovulum*) are indicated where appropriate. 

A novel *Rhodobacter* species, designated strain M37P, was isolated in 2006 by RFR from the Mushroom hot spring runoff within the Lower Geyser Basin of Yellowstone National Park. The isolate was purified using aerobic *Thermus* medium with rifampin [29]. A partial 16S rRNA (470 bp) construct of the pure culture was sequenced (GenBank accession DQ363135), and identified “*Rhodobacter gluconicum*” as the closest relative at that time (97% identity; 451/466 bp), which unfortunately is not available in culture collections and a description has not been published. Since the initial isolation, several new species have come to light that share an even closer relationship. Due to its unique habitat and the fact that very little is known about this new isolate, we decided to sequence its genomic DNA and compare it to the better characterized *Rhodobacter* species.

## 2. Materials and Methods

### 2.1. Strain Isolation 

The M37P strain was originally isolated from a runoff channel, at a 37 °C temperature sampling point, which flowed from Mushroom Spring, a slightly alkaline (pH 8.0–8.2) hot spring in the lower geyser basin of Yellowstone National Park (US). The strain was labeled M37P, for Mushroom Spring, 37 °C Pink-colored isolate. The original isolate was grown aerobically on Thermus medium [29] containing 0.1% yeast extract and 0.1% tryptone in Castenholz salts (pH 7.6–7.8). Cultures were grown on agar plates incubated at 37 °C and allowed to grow for 2–3 weeks. Pinkish-red colonies were picked and consecutively transferred to new plates three times to obtain pure cultures and subsequently used to inoculate liquid medium.

### 2.2. Cultivation

Subsequent growth was also conducted in Thermus medium containing 0.3% yeast extract, 0.3% tryptone, and 0.3% casamino acids (final pH 7.6–7.8). Increasing the concentration of yeast extract, tryptone, or casamino acids above the 0.3% level did not increase the growth rate or final cell yield. Metabolism of malate, succinate, glutamate, and glucose for growth was tested in Thermus medium [29] and in two different minimal media, Minimal 22 and RCVB [30], at concentrations of 30 mM. Growth factor requirements were tested for thiamin (1 mg/L), nicotinic acid (1 mg/L), and biotin (15 µg/L) in RCVB medium. Growth was tested in 50–100 mL liquid cultures and agar plates, under aerobic and anaerobic conditions, both in light and dark conditions for several weeks. The growth temperature range tested in shaking liquid cultures was 30–45 °C. The pH range tested was from 5–10 in liquid Thermus medium containing 0.3% yeast extract, 0.3% tryptone, and 0.3% casamino acids. No growth was observed in any of the anaerobic conditions. Oxidase activity was tested using BBL DrySlides from Becton Dickinson and catalase activity was tested using 3% (*w*/*v*) H_2_O_2_ by assessing bubble production as a positive result. Cells were observed using a MEIJI Techno (Chikumazawa, Japan) MT4200H microscope equipped with brightfield and phase contrast condenser and images were obtained with an attached Motic digital camera (Motic North America, Richmond, BC, Canada). 

Pigments were extracted from dark and light incubated cultures using a methanol extraction from 50 mL aerobically grown cultures. Cells were pelleted by centrifugation (5 min at 10,000× *g*) and resuspended in 5 mL methanol, after which they were centrifuged again and the supernatant was used for spectral analysis. Absorption spectra were obtained using an Evolution 300 UV-vis spectrophotometer (Thermo Scientific, Waltham, MA, USA) with the VisionPro software (version 4.5.0). 

### 2.3. Genome Sequencing

We isolated gDNA of the M37P isolate from living cultures with the GeneJET DNA purification kit (Thermo Scientific). The cells form a visible capsule and cluster together during cultivation, which complicated lysis with the standard protocol. An additional cell lysis step in 5% SDS and proteinase K (1 mg/mL) was needed, and incubation at 65 °C was performed for several hours to assure sufficient lysis of the cells. The quality and quantity of purified DNA were determined using Qubit and NanoDrop and showed a concentration of 21 ng/µL and an A_260_/A_280_ absorbance ratio of 1.73, which indicated that there was sufficient high-quality DNA for sequencing. The DNA library was prepared following the Nextera DNA Flex library prep kit instructions (Illumina, San Diego, CA, USA) using 300 ng of genomic DNA. The genome was sequenced by an Illumina MiniSeq system with 500 µL of a 1.8-pM library, using paired-end sequencing (2 × 150 bp). Quality control of the reads was performed using FASTQC within BaseSpace (Illumina; version 1.0.0), using a k-mer size of 5 and contamination filtering. De novo assembly of the data was performed using SPAdes (version 3.10.0) [31] through PATRIC [32]. The assembled genome had a coarse consistency of 98.5% and fine consistency of 97.5% and was considered of good quality. The genome sequence was annotated using the Rapid Annotations using Subsystems Technology (RAST) server (version 2.0) [33]. Completeness and possible contamination were assessed with CheckM [34]. 

### 2.4. Whole Genome Comparison

The average percentage nucleotide identity (ANI) was calculated using JSpecies [35], which uses a pairwise genome comparison algorithm to measure probability if two or more genomes belong to the same species. The compared genomes were either selected from the curated reference database GenomesDB (by JSpecies) or uploaded from GenBank for those not present in the JSpecies database. Reciprocal average amino acid identity (AAI) comparison was calculated using the AAI calculator from the Konstantinidis Lab (http://enve-omics.ce.gatech.edu/aai/index; accessed on 1 April 2022) [36]. The following alignment options were used: minimum length, zero aa; minimum identity, 20%; minimum alignments. 50.

A whole genome-based phylogenetic tree was generated using the CodonTree method within PATRIC [32], which used PGFams as homology groups. Representative genomes from different *Rhodobacter* species were selected for the analysis. The number of single-copy genes from PGFams was set at 1000, and 557 PGFams were found among the selected genomes using the CodonTree analysis. Protein sequences were aligned using MUSCLE [37], and the nucleotide coding gene sequences were aligned using the Codon_align function of BioPython. The aligned proteins and coding DNA from single-copy genes were used for RaxML analysis [38,39] within PATRIC [32]. iTOL was used to draw the phylogenetic trees expressed in the Newick phylogenetic tree format [40].

### 2.5. Single-Gene Phylogenetic Comparisons

The multiple sequence alignments for the 16S rRNA and xanthorhodopsin comparisons were performed using ClustalW [41]. For the 16S rRNA analysis, the phylogenetic tree was designed by using the maximum likelihood method and general time reversible model [42]. The tree with the highest log likelihood was used. Initial tree(s) for the heuristic search were obtained automatically by applying Neighbor-Join and BioNJ algorithms to a matrix of pairwise distances estimated using the maximum composite likelihood (MCL) approach, and then selecting the topology with superior log likelihood value. A discrete gamma distribution was used to model evolutionary rate differences among sites (5 categories (+G, parameter = 0.8636)). The rate variation model allowed for some sites to be evolutionarily invariable ([+I], 80.81% sites). 

For the xanthorhodopsin tree, the evolutionary history was inferred by using the maximum likelihood method and Le_Gascuel_2008 model [43]. The tree with the highest log likelihood (−7279.39) was used. Initial tree(s) for the heuristic search were obtained automatically by applying Neighbor-Join and BioNJ algorithms to a matrix of pairwise distances estimated using the JTT model, and then selecting the topology with superior log likelihood value. A discrete gamma distribution was used to model evolutionary rate differences among sites (5 categories (+G, parameter = 1.1987)). The rate variation model allowed for some sites to be evolutionarily invariable ([+I], 14.31% sites).

In both cases, 500 bootstrapping rounds were used to construct the tree. The final trees are drawn to scale, with branch lengths measured in the number of substitutions per site. The 16S rDNA analysis involved 29 sequences, derived from whole genomes where available, with the following PATRIC-BRC accession numbers: fig|2715277.3.rna.28 (*Rhodobacter* sp. M37P); fig|2883999.5.rna.51 (*Rhodobacter* sp. Har01); fig|2807096.3.rna.45 (*Rhodobacter* sp. N10); fig|2852097.3.rna.48 (*Rhodobacter* sp. HSP-20); fig|1985673.3.rna.53 (*Rhodobacter* sp. CCP-1); fig|2593022.3.rna.42 (*Rhodobacter* sp. SYSU G02214); fig|2593021.3.rna.42 (*Rhodobacter* sp. SYSU G03088); fig|2593019.3.rna.27 (*Rhodobacter* sp. SYSU G02092); fig|2593020.3.rna.27 (*Rhodobacter* sp. SYSU G02094); fig|2562317.3.rna.48 (*Rhodobacter* sp. SY28-1); fig|2528036.3.rna.32 (*Rhodobacter* sp. YIM 73028); fig|2249421.3.rna.20 (*Rhodobacter* sp. YIM 73036); fig|1063.35.rna.41 (*Rhodobacter sphaeroides* SCJ); fig|1063.34.rna.43 (*Rhodobacter sphaeroides* FY); fig|39723.4.rna.7 (*Rhodobacter sphaeroides* f. sp. denitrificans IL106); fig|272943.71.rna.1 (*Rhodobacter sphaeroides* 2.4.1); fig|1061.9.rna.57 (*Rhodobacter capsulatus* DSM 1710); fig|1054202.4.rna.47 (*Rhodobacter viridis* JA737); fig|1188250.3.rna.48 (*Rhodobacter blasticus* DSM 2131); fig|1185920.4.rna.50 (*Rhodobacter veldkampii* DSM 11550); fig|445629.8.rna.51 (*Rhodobacter johrii* JA192); fig|43057.6.rna.48 (*Rhodobacter azotoformans* KA25); fig|1063.31.rna.46 (*Rhodobacter blasticus* 28/5); fig|1188249.3.rna.49 (*Cereibacter changlensis* JA139); fig|446682.3.rna.35 (*Rhodobacter maris* JA276); fig|439529.3.rna.19 (*Rhodobacter ovatus* JA234); fig|407234.3.rna.49 (*Rhodobacter vinaykumarii* DSM 18714); fig|453582.3.rna.47 (*Rhodobacter aestuarii* DSM 19945); fig|1884310.3.rna.15 (*Rhodobacter* sp. 24-YEA-8); fig|272942.6.rna.3 (*Rhodobacter sediminis* SB 1003). 

The xanthorhodopsin analysis used 31 protein sequences with the following accession numbers: LGHU01000114 (*Pseudorhodobacter antarcticus* KCTC 23700); 1081.4.con.0047 (*Rhodospirillum sodomense*); ATUR01000008 (*Sphingopyxis baekryungensis* DSM 16222); JASG01000004 (*Sulfitobacter guttiformis* KCTC 32187); JEMV01000009 (*Sphingobium* sp. Ant17); JPPQ01000083 (*Sphingobium* sp. ba1); 204455.19.con.0021 (*Rhodobacter* sp. M37P); NZ_DS990628 (*Octadecabacter arcticus* 238); NC_015581 (*Thioalkalimicrobium cyclicum* ALM1); LAPV01000235 (*Devosia psychrophila* Cr7-05); 85076.5.con.0174 (*Marichromatium* SE5_Pink); CP006644 (*Sphingomonas sanxanigenens* DSM 19645); MBFX01000002 (*Spiribacter salinus* M7-304); BMGD01000001 (*Blastomonas aquatica* CGMCC 1.12851); CP042288 (*Aquisalinus flavus* D11M-2); PDEQ01000001 (*Longibacter salinarum* KCTC 52045); RAHX01000001 (*Erythrobacter aquimixticola* JSSK-14); NTKP01000020 (*Rhodothermaeota* MED-G12); RZHG01000028 (*Halomonas* sp. DSM 19434); SJDN01000025 (*Tabrizicola* sp. WMC-M-20); VHHQ01000006 (*Thermus thermophilus* IB-21); NC_007677 (*Salinibacter ruber* DSM 13855); BJXV01000002 (*Halovibrio variabilis* NBRC 102410); CP011494 (*Marinobacter psychrophilus* 20041); LN681225 (*Legionella hackeliae* ATCC35250); MTEJ01000043 (*Thiothrix lacustris* A8); VMKO01000001 (*Halopeptonella vilamensis* DSM 21056); DSHZ01000261 (*Thermus islandicus* SpSt-189); NCEQ01000006 (*Brevundimonas subvibrioides* 32-68-21); RWJI01000002 (*Sphingorhabdus wooponensis* 03SU3-P); AXCW01000007 (*Actinotalea ferrariae* CF5-4) 

In both phylogenetic trees, all positions with less than 95% site coverage were eliminated, i.e., fewer than 5% alignment gaps, missing data, and ambiguous bases were allowed at any position (partial deletion option). Evolutionary analyses were conducted in MEGA X [44], and tree visualization was performed in iTOL [40].

### 2.6. Structure Modeling

The xanthorhodopsin protein sequence was obtained from the M37P genome and used for 3D modeling using SWISS-MODEL (https://swissmodel.expasy.org; accessed on 9 April 2022) [45]. The SWISS-MODEL template library (SMTL version 12 January 2022, PDB release 7 January 2022) was searched with BLAST [46] and HHblits [47] for evolutionary related structures. The target sequence was searched in the BLAST database against the primary amino acid sequence contained in the SMTL. A total of 21 templates were found. An initial HHblits profile was built using the procedure outlined in [47], followed by 1 iteration of HHblits against Uniclust30 [48]. The obtained profile was then searched against all profiles of the SMTL. A total of 191 templates were found. The best matching target was found to be 6nwd (GII198protein; rhodopsin derived from *Gloeobacter violaceus* PCC 7421) with 45.5% sequence identity, which was used for model construction. Models are built based on the target–template alignment using ProMod3 [49]. Coordinates that are conserved between the target and the template are copied from the template to the model. Insertions and deletions are remodeled using a fragment library. Side chains are then rebuilt. Finally, the geometry of the resulting model is regularized by using a force field. The global and per-residue model quality has been assessed using the QMEAN scoring function [50]; a QSQE score of 0.18 and coverage of 0.98 was obtained.

## 3. Results and Discussion

### 3.1. Genome Analysis

Sequencing the *Rhodobacter* sp. M37P genome generated 5,051,274 reads, yielding a total of 420.96 Mbp of sequencing data. The assembled genome consisted of 59 contigs, with the largest contig being 483,911 bp, with a N_50_ of 255,108 bp. The GC content was 67.5% and the length of the M37P genome was 3.63 Mbp. The genome sequence was annotated using the RAST server, which identified 3732 coding sequences (CDS) and 46 tRNAs. There were also 56 short repeat regions. A CheckM analysis showed the genome to have 100% completeness and no contamination. A comparison of genome properties with related *Rhodobacter* species is provided in Table 1. The GC content and genome size are similar to those of *Rhodobacter blasticus* (now *Fuscovulum blasticum*) DSM 2131 (66.5% and 3.58 Mbp), *Rba. capsulatus* DSM 1710 (66.5% and 3.67 Mbp), and slightly lower than *Rba.* (now *Luteovulum*) *sphaeroides* 2.4.1 (68.7% and 4.60 Mbp). The M37P genome sequence contained genes for nitrogen fixation (nitrogenase and nitrogenase reductase) and sulfur oxidation (*Sox*XYZ), which is consistent with *Rhodobacter* metabolism. 

**Figure 1:**
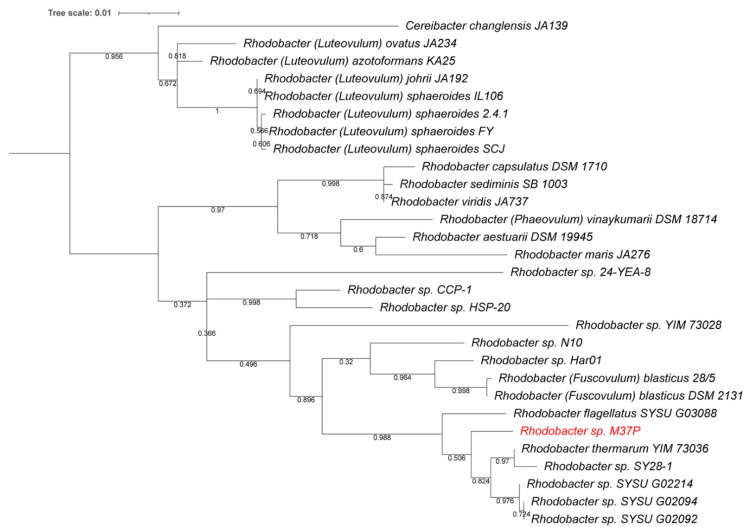
16S rRNA-derived phylogenetic tree for *Rhodobacteraceae* species. The phylogenetic tree was calculated in MegaX and iTOL was used to draw the phylogenetic trees expressed in the Newick phylogenetic tree format. The length of the branches is proportional to the number of nucleotide substitutions per site, and bootstrap values were generated from 500 bootstrapping rounds. The new isolate, *Rhodobacter* strain M37P, 16 rRNA is indicated in red.

A BLAST (NCBI) comparison using the complete 16S rRNA from our new species indicated *Rba. thermarum* YIM73036 and *Rba. blasticus* DSM 2131 as closest relatives: *Rba. thermarum* YIM73036 97.74% (1429/1462 bp) and *Rba. blasticus* 28/5 97.08% (1430/1473 bp). Using the complete 16S rRNA, the M37P isolate is only 96% (1368/1420 bp) identical to “*Rhodobacter gluconicum*”, to which it was previously compared. These values are below the proposed species delineation for 16S rRNA comparisons of 98.7% [52]. A phylogenetic tree based on the 16S rRNA sequences of related *Rhodobacter* species is provided in Figure 1. This shows *Rba. thermarum* YIM73036 and *Rba. flagellatum* SYSU G03088 as the closest relatives, but clearly separated from the new strain M37P on the tree. All three appear to be part of a separate clade on the tree, which also includes some newer strains SY28-1, SYSU G02214, SYSU G02094, and SYSU G02092. These latter strains have not been formally described, but their genomes are available in the GenBank database. Interestingly, all of the SYSU strains were isolated from hot spring sediments in Tibet. The full-length M37P 16S rDNA sequence, derived from the genome, was submitted to GenBank with accession number ON328317. 

A whole genome-based JSpecies comparison [35] of the average percentage nucleotide identity (ANI) between strain M37P and published *Rhodobacter* genome sequences deposited in GenBank, showed the highest identities with *Rba. thermarum* YIM73036, (87.5%), *Rba. flagellatus* SYSU G03088 (85.2%), *Rba*. strain SY28-1 (82.6%), *Rba*. strain YIM73028 (80.1%), and *Rba. blasticus* DSM 2131 (76.1%). At well below the 95% cutoff for same species, strain M37P is clearly a distinct species in its own right. All other *Rhodobacter* species showed identities at or lower than 74%. 

A reciprocal average amino acid identity (AAI) comparison showed an 84.1% identity (SD 13.7%; from 3441 proteins) with *Rba. thermarum* YIM73036, 83.2% (SD 13.2%; from 4030 proteins) with *Rba.* sp. 28-1, 82.7% (SD 13.4%; from 3419 proteins) with *Rba. flagellates*, and 72.6% (SD 14.9%; from 2498 proteins) with *Rba. blasticus*. These are all below the arbitrary species delineation of 85–90% AAI, and further defines the species individuality of the M37P isolate.

**Figure 2:**
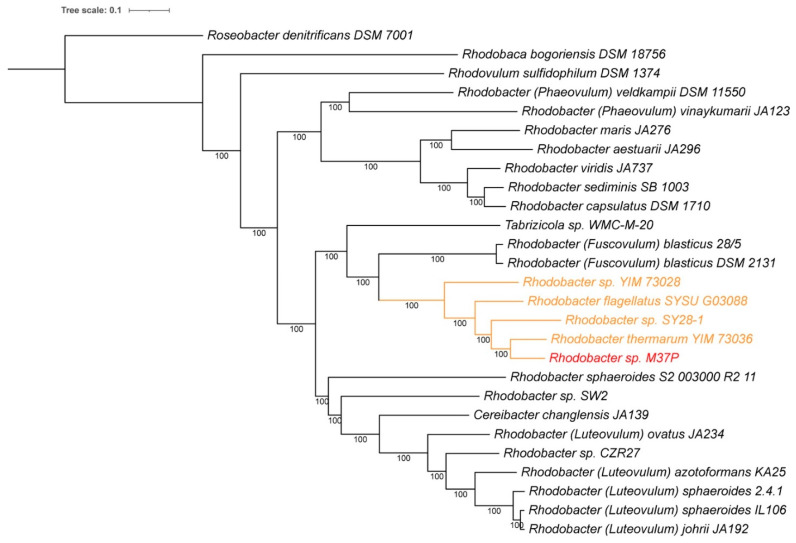
Whole genome-based phylogenetic tree of all sequenced *Rhodobacter* species. *Roseobacter denitrificans* was used as an outgroup and *Rhodovulum sulphidophilum* and *Rhodobaca bogoriensis* were included for comparison. One hundred rounds of the ‘rapid bootstrapping’ option of RaxML were used to generate the support values for the phylogenetic tree. The branch length tree scale is defined as the mean number of substitutions per site, which is an average across both nucleotide and amino acid changes. Genome accession numbers can be found in Table 1. The *Rhodobacter calidifons* M37P genome is indicated in red and species with ANI > 80% to M37P are colored orange.

A whole genome-based phylogenetic analysis of the sequenced *Rhodobacter* genomes constructed using RaxML [38,39], is consistent with the ANI and 16S rRNA analysis presented above. As can be seen in Figure 2, the *Rhodobacter* sp. M37P genome is clearly different from any of the other *Rhodobacter* species, and part of a clade of five species (orange in Figure 2) that includes the recently discovered *Rba. thermarum* strain YIM 73036, strain SY28-1, *Rba. flagellatus* SYSU G03088, and strain YIM73028. Except for *Rba. thermarum* and *Rba. flagellatus*, none of these new strains have been fully characterized; however, at least three of the four appear to be isolated from hot spring sediments, similar to M37P. Most closely related is the recently discovered *Rba. thermarum* YIM73036, which can grow at a maximum temperature of 55 °C, although 37–45 °C is optimum [17]. Most *Rhodobacters* grow best at 30–37 °C (Table 1).

### 3.2. Species and Growth Characteristics

**Figure 3:**
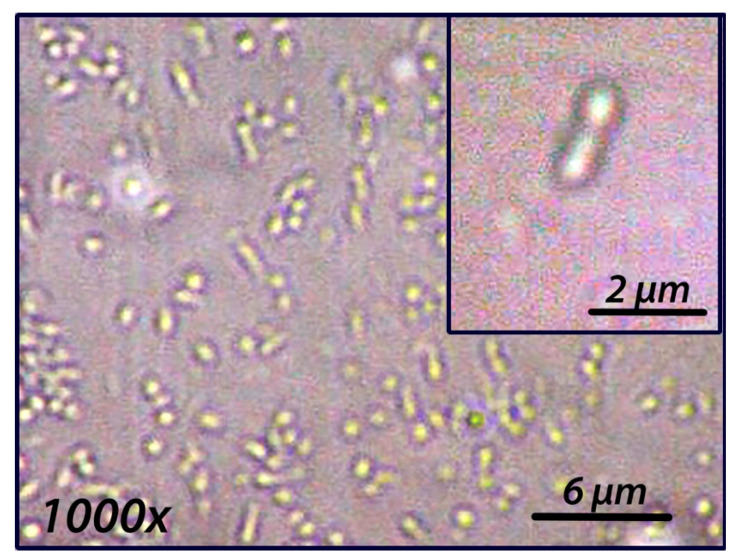
Phase contrast microscopy of *Rhodobacter* strain M37P at 1000× total magnification. Insert is 2.5× digitally magnified and contrast autocorrected.

Microscopic examination of the purified strain (Figure 3) showed that cells are rod-shaped, 1 to 2 μm long and 0.5 to 0.7 μm wide. Multiplication appears to occur by binary fission and cells appear to be motile. Interestingly, cells appear to exhibit a directional twitching motility towards the light in glass petri dishes (data not shown). Strain M37P was found to be catalase and oxidase positive. The growth temperature range tested in shaken cultures was 30–45 °C, with an optimum temperature of 38–39 °C and doubling time of 8–9 h. However, the doubling time of M37P on minimal medium is very long (14–20 h under our tested conditions). The temperature optimum of *Rba.* M37P is slightly higher than most *Rhodobacter* species, but is closer to the temperature optimum of the closely related *Rba. thermarum* YIM73036 (Table 1). The optimal pH range was found to be 7.6 to 8.0. 

Like other *Rhodobacter* isolates, we found that M37P will use malate, succinate, glutamate, and glucose for growth supplementation in Thermus medium and in minimal medium (Minimal 22 and RCVB). Many photosynthetic *Rhodobacter* species are able to grow both aerobically and anaerobically. In our growth studies, using both rich nutrient broth or minimal RCVB medium, we were unable to grow *Rhodobacter* sp. M37P under anaerobic conditions in either liquid medium or on plates. This is not typical for *Rhodobacter* species; however, aerobic anoxygenic phototrophs (AAPs) on the other hand are unable to use bacteriochlorophyll for anaerobic growth, but they do perform cyclic photophosphorylation only in the presence of oxygen [53]. AAP lack the RuBisCo enzyme for CO_2_ fixation and are therefore not autotrophic [54]. Indeed, when checking the genome of M37P we found that it does contain *puf* genes for the photosynthetic reaction center subunits L, M, and H, but does not contain the RuBisCo gene for fixation of CO_2_. RuBisCo is an essential protein in the Calvin–Benson–Bassham cycle that catalyzes the addition of carbon dioxide to ribulose-1,5-bisphosphate. The presence of multiple RuBisCo genes is not uncommon in Proteobacteria [55,56,57,58]. In the same species, a single RuBisCo gene can be present (Form II), or alternatively, a two-subunit form of RuBisCo (Form I) consisting of large (*rbc*L) and small (*rbc*S) subunits, or both forms can be found as well [57]. Several of the *Rba. sphaeroides* and *Rba. capsulatus* contain both versions of the rubisco genes (Table 1). However, neither the small or large RuBisCo subunit, nor the single Form II RuBisCo was found annotated in the M37P genome. We also performed a BLAST search within PATRIC using all three of the *Rba. sphaeroides 2.4.1.* RuBisCo genes to see if it may have not been annotated correctly in the automated annotation, but again no rubisco genes were found in the strain M37P genome. Only a single gene encoding a protein ‘similar to a RuBisCo type III’ was found annotated. An NCBI-BLAST search of this gene identified it as a RuBisCo-like protein, not involved in CO_2_ fixation but playing a role in an uncharacterized sulfur salvage pathway and has been shown to catalyze a novel isomerization reaction of 5-methylthio-D-ribulose 1-phosphate in *Rhodospirillum rubrum* [59].

The lack of anaerobic growth, the presence of *puf* genes, and absence of *rbc* genes indicate that strain M37P is an AAP, which is the first AAP identified in the genus *Rhodobacter*. *Roseobacter denitrificans* Och114 is the closest known AAP relative to *Rhodobacter*, based on 16S rDNA taxonomic analysis [53]. We included *Rsb. denitrificans* Och114 in our whole genome-based phylogenetic tree comparison (Figure 2). This shows the closer relationship of M37P to other *Rhodobacter* species than to *Roseobacter denitrificans*, consistent with the earlier identification of M37P as a *Rhodobacter* species. Several of the other species on the M37P clade are also lacking all of the RuBisCo genes (Figure 2 and Table 1). Unfortunately, not all of them have been characterized; however, only weak anaerobic photo-heterotrophic growth was reported for *Rba. thermarum*, while no photolithoautotrophic growth was observed [9], which is consistent with the lack of RuBisCo. Further comparative studies will be necessary to determine whether other members of this clade are also AAPs. 

**Figure 4:**
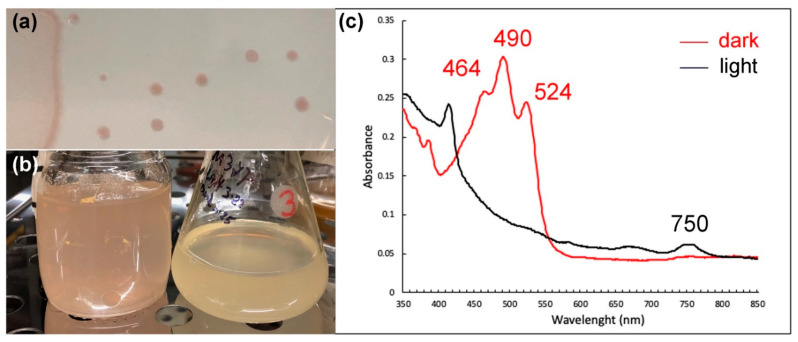
(**a**) Colonies of *Rhodobacter* sp. M37P grown on dark-incubated Thermus medium plates. (**b**) Dark- (left) and light- (right) incubated aerobic liquid cultures of strain M37P. (**c**) Absorption spectra of extracted pigments from dark-incubated (red) and light-incubated (black) aerobic *Rhodobacter* sp. M37P cultures. Spectral peak maxima are indicated.

The pigmentation of M37P is a pinkish, red-centered colony with a lighter edge and the red center becomes more intense upon continuous aerobic growth. Interestingly, the pinkish-red pigment that is present in the culture under aerobic dark conditions, disappears under light conditions, and returns when the cultures are subsequently incubated in the dark. This is different from the aerobic repression of photosynthetic pigment synthesis observed in other *Rhodobacter* species [60,61]. On the other hand, in aerobic anoxygenic phototrophic bacteria, the influence of light intensities was found to inhibit bacteriochlorophyll a (Bchl *a*) and carotenoid synthesis [53,62,63]. A two- to three-fold increase in the concentration of Bchl *a* has been observed in dark-incubated periods, for example, in the AAPs *Erythrobacter hydrolyticum, Erythromicrobium sibiricum*, and *Roseobacter denitrificans* [62,63,64]. The light-dependent regulation of carotenoids appears to be different in various AAPs, where carotenoids are greatly inhibited by blue light in *Roseobacter denitrificans*, but not affected by light of any wavelength in *E. ramosum* [53,63]. Dark-incubated whole cell absorption spectra of *Rba.* strain M37P showed maxima characteristic of BChl *a* (590, 795 and 862 nm). After performing methanol-based pigment extractions of aerobic dark and light cultures of M37P, we observed a small peak at 750 nm, possibly due to bacteriopheophytin (Figure 4, black spectrum). However, in the dark-incubated cultures, there was a high abundance of pigments with peaks at 464 nm, 490 nm, and 524 nm (red spectrum in Figure 4), which were not seen in cultures that were incubated in the light for several hours (black spectrum; Figure 4). A similar light-induced washout of carotenoid pigments was observed in *Rsb. denitrificans* [63]. This indicates that there is a similar regulation of carotenoid synthesis in M37P as in *Rsb. denitrificans*. Compared to anaerobic purple non-sulfur phototrophs, AAP cells typically contain only small amounts of BChl relative to the abundance of carotetoids (about 1:10 ratio [53,62]). This also appears to be the case in M37P, based on the spectra of the extracted pigments (Figure 4), further indicative of the fact that strain M37P is an AAP.

Comparison of the photosynthetic systems of strain M37P and the other *Rhodobacter* genomes also revealed that this strain is lacking the genes for the light harvesting complex II (LHII), which is typically an important photosystem component in *Rhodobacter* (and other photosynthetic bacteria), and augments the collection of solar energy [65,66]. The LHII antenna complexes are peripheral complexes that transfer energy to the LHI complexes, which in turn transfer energy to the reaction centers (RCs) embedded therein [67]. The LHII alpha and beta subunit genes (*pucA* and *pucB*) are typically found in the *pucBACDE* gene cluster. PucC proteins have been implicated in LH1 and LH2 assembly and are part of the bacteriochlorophyll delivery (BCD) family of the major facilitator superfamily [68,69].

All of the *Rhodobacter* genomes have a *pucC* gene homolog, including strain M37P, that is clustered with the reaction center H subunit gene in their genome; however, the second *pucC* homolog, and in fact the entire *pucBACDE* gene cluster that encodes the LHII complex genes, is lacking from strain M37P, and from strains YIM73028, SYSU G03088, SY28-1, and YIM73036 (Table 1), all of which form the unique clade with strain M37P (Figure 1 and Figure 2). None of these genes were found annotated in these genomes and a BLAST search in PATRIC using the *puc* genes from the *Rba. sphaeroides*, *Rba. capsulatus*, and *Ceribacter changlensis* LHII gene cluster did not find any *pucBACDE* gene homologs for LHII complexes in any of the members of the M37P clade. 

A difference in diversity of LH systems in AAPs as compared to analogous complexes from anaerobic phototrophic bacteria has been observed earlier [53,70]; however, the closest AAP relative of M37P, *Rsb. denitrificans*, does produce a functional peripheral LHII complex. On the other hand, some other AAPs, *Acidiphilium rubrum* and *Porphyrobacter* also appear to lack LHII complexes [71,72]. The lack of LHII or the reduced size of the complex is consistent with a more auxiliary role of phototrophy in AAPs [70]. 

Although the evolution of phototrophy in Proteobacteria is a complex issue, it has been proposed that AAPs have evolved from anaerobic anoxygenic phototrophs [53,73]. This was mainly hypothesized based on earlier DNA–DNA hybridizations of AAPs and the fact that anoxygenic phototrophy evolved well before the gradual oxygenation of the Earth [74,75]. It is therefore intriguing to speculate that *Rba. calidifons* M37P has evolutionary lost the ability for anaerobic photosynthesis and autotrophic growth, given its close phylogenetic relationship to the anaerobic phototrophic *Rhodobacter* species. However, at this point, it is not certain if the evolution to an AAP would have occurred by the loss of carbon fixation genes only, or what specifically causes the loss of ability for anaerobic photosynthesis. The lack of a LH II complex certainly indicates that there is a more complex evolutionary story involved. A more detailed phylogenetic and biochemical analysis of the phototrophy of *Rhodobacter* M37P and other related AAPs will be necessary to determine whether this occurred by evolutionary gene loss from anaerobic photosynthetic *Rhodobacter* species, or if there is an earlier divergence in evolution that formed the M37P containing clade. 

### 3.3. Xanthorhodopsin

Upon further comparison of the *Rba.* M37P genome to 75 other *Rhodobacter* genomes (Supplementary Table S1), we found that it contains a unique gene for xanthorhodopsin (XR) (PGFam_08288266; locus tag: G8O29_16735). Xanthorhodopsins are light-harvesting proton pumps with a dual chromophore [76,77,78]. They have one retinal molecule, similar to archaeal bacteriorhodopsin (BR) and the more widespread proteorhodopsin (PR); in addition, XR contains an additional carotenoid antenna chromophore that allows the cells to utilize a wider range of light for energy conversion. Other than strain M37P, none of the analyzed *Rhodobacter* genomes contain any form of rhodopsin gene, nor have any rhodopsin-containing *Rhodobacter* species been described to our knowledge. To identify the closest relatives of the *Rhodobacter* XR (RXR), we searched all PATRIC genomes (derived from NCBI GenBank) and performed a phylogenetic comparison of the translated protein to other annotated xanthorhodopsins. 

**Figure 5:**
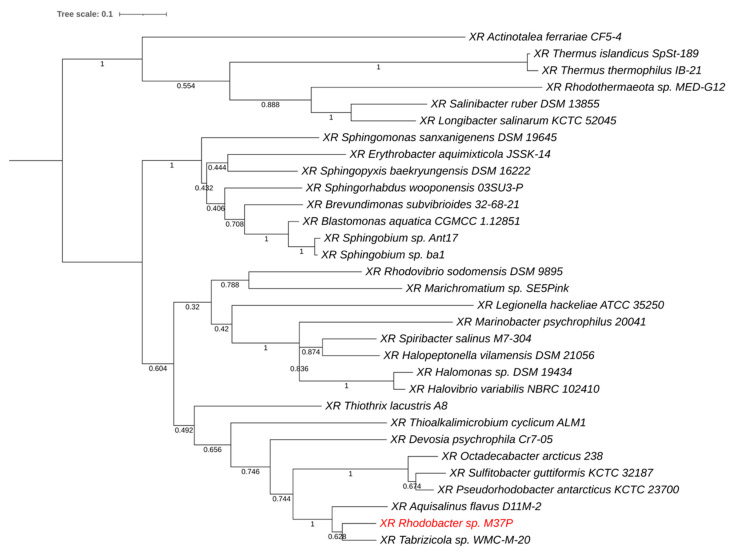
Phylogenetic comparison of xanthorhodopsin from *Rba.* sp. M37P to its closest xanthorhodopsin homologs derived from genome sequences. The M37P XR protein (in red) forms a separate clade with closely related XRs from *Aquisalinus flavus* D11M-2 and *Tabrizicola* sp. WMC-M-20.

Figure 5 provides a phylogenetic tree of the closest homologs. M37P xanthorhodopsin (labeled in red) forms a separate clade with closely related XRs from *Aquisalinus flavus* D11M-2 and *Tabrizicola* sp. WMC-M-20. The homology with these proteins was 82% (212/257 aa.) for *Aqs. flavus* and 86% (222/258 aa.) for *Tabrizicola sediminis* WMC-M-20. *Aquisalinus flavus* D11M-2 belongs to the *Parvularculaceae* and was isolated from the saline Lake Dasugan in China and is strictly heterotrophic and aerobic [79]. *Tabrizicola sediminis* WMC-M-20 is an AAP isolated from a saline lake in Tibet [80] and belongs to the larger family of *Rhodobacteriaceae*. 

The xanthorhodopsin gene in strain M37P is followed by several carotenoid biosynthesis genes (polyprenyl synthetase; phytoene desaturase (neurosporene-forming) (EC 1.3.99.28); phytoene synthase (EC 2.5.1.32); lycopene beta-cyclase (EC 5.5.1.19); and 15,15′-beta-carotene monooxygenase). The rhodopsin retinal chromophore is an oxidative cleavage product of beta-carotene, which is catalyzed by a beta-carotene 15,15′-monooxygenase [81,82]. The presence of the retinal biosynthetic enzyme, beta-carotene 15,15′-monooxygenase, indicates that strain M37P is capable of producing a functional rhodopsin. Three of the other carotenoid biosynthetic enzymes located in between—polyprenyl synthetase, phytoene desaturase, and phytoene synthase—are known to catalyze the formation of neurosporene and lycopene carotenoids all the way from the terpenoid backbone synthesis (Supplementary Figure S1) [83]. Lycopene beta-cyclase is involved in several downstream carotenoid modification pathways, including 7,8-dihydroxy-beta-carotene, beta-carotene, lutein and neurosporaxanthin synthesis [84] (Supplementary Figure S1), and is likely important for the synthesis of carotene or the additional carotenoid that is associated with functional xanthorhodopsins. This indicates that all the genes needed for the biosynthesis of beta-carotene and a functional xanthorhodopsin protein are present in the *Rhodobacter* M37P genome. This synteny is also conserved in the *Tabrizicola* sp. WMC-M-20 and several of the other xanthorhodopsin-containing genomes, which supports this hypothesis. 

Proton-pumping rhodopsins consist of seven transmembrane helices that pump protons along the cell membrane and thereby create a proton gradient that is used for ATP production, thereby providing a simple method for light to biological energy conversion. 

Bacteriorhodopsin is only found in Archaea, whereas Proteorhodopsin is now found to be widespread amongst marine members of the Bacteria and Archaea [85,86]. On the other hand, only a limited amount of xanthorhodopsins have been described. XR has some unique structural differences with other rhodopsins, such as BR and PR [78,87,88]. XR-type rhodopsins have an additional binding pocket for the carotenoid antenna molecule, and share the structural features of having elongated helices, a flipped B–C loop and a 3-omega motif [78]. To confirm that the *Rba.* sp. M37P gene encodes a true xanthorhodopsin, we created a 3D structural model of RXR using SWISS-MODEL and analyzed it for the potential presence of these distinguishing features. 

**Figure 6:**
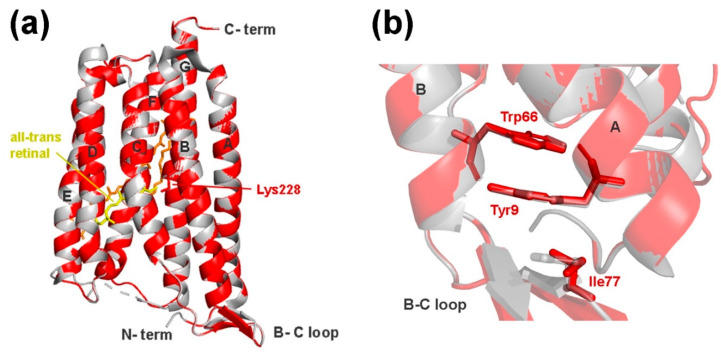
(**a**) Superimposed structures of *Rba.* sp. M37P xanthorhodopsin (RXR; red) and the *Salinibacter ruber* xanthorhodopsin (light gray, PDB entry 3DDL) The retinal and salinixanthin chromophores from XR are labeled in yellow and orange, respectively. (**b**) Detailed structural image of the 3-omega motif formed by pi-stacking of aromatic residues in helix A, B, and the B–C loop. RXR residues are labeled in red and *Salinibacter* XR residues shown in gray.

The RXR structure has seven transmembrane helices, similar to other microbial rhodopsins, and has Lys228 positioned correctly for the Schiff base binding of the retinal chromophore (Figure 6A). A superposition of RXR with other rhodopsin structures shows that it is more similar to XR than BR or PR, as expected from the sequence similarity. The following RMSD (root-mean-square deviation) values were obtained in PyMOL superpositions: 0.090 with XR (1418 atoms) from *Salinibacter ruber* (PDB entry 3DDL); 1.714 with GR (1541 atoms) from (PDB entry 6NWD); 2.010 with BR (954 atoms) from *Halobacterium salinarum* (PDB entry 1C3W); and 3.002 with PR (1187 atoms) from an uncultured gammabacteria (PDB entry 4KLY). The superposition of RXR with *S. ruber* XR (Figure 6) shows that it also has the typical elongated A, B, and G helices and is capable of forming the characteristic β-sheet structure in the BC loop, which is not seen in PR or BR structures. The binding pocket for the carotenoid antenna molecule is also present in the RXR structure, indicating that it is capable of binding a second chromophore (both retinal and salinixanthin were modeled into the structure in Figure 6). 

The eubacterial rhodopsin structures also share a 3-omega motif, formed by three non-consecutive aromatic amino acid residues from the extended helices and the B-C loop structure, which are linked through aromatic pi-stacking interactions. The RXR structure also positions Tyr9 (helix A; equivalent to Tyr13 in XR) and Trp66 (helix B; equivalent to Trp70 in XR) in such interactions; however, the third position is substituted by a non-aromatic Ile77 residue (instead of Tyr81 in XR) (Figure 6B). The 3-omega motif is conserved in all different eubacterial rhodopsin pumps, but completely lacking from archaeal BR-pumps, and is thought to be involved in structural interprotomer interactions, rather than being correlated to a specific function [78]. The potential lack of the third pi-stacking aromatic interaction in the RXR B–C-loop, and its possible structural implications will need further study. Nevertheless, the sequence homology and structural comparison indicate that the rhodopsin gene found in strain M37P indeed encodes a xanthorhodopsin. 

Interestingly, the absorption spectra of the extracted pigments (Figure 4) correspond well with the major absorption spectra described for the xanthorhodopsin carotenoid component [76,77,78,89], where absorption spectra at 521 nm, 487 nm and 456 nm are due to structurally bound salinixanthin. Further analysis of the purified holo-protein and HPLC analysis of the M37P carotenoids will be necessary to determine its characteristics; however, based on our current analysis of this unique strain, the pinkish-red pigmentation observed in dark-adapted cultures is due to the production of carotenoids, possibly linked to a functional xanthorhodopsin. 

### 3.4. Xanthorhodopsin in Mushroom Spring Microbial Mats

The potential importance of rhodopsins in the energy metabolism of the Mushroom Spring microbial ecosystem has been mentioned earlier during a metagenomic analysis of the microbial mat communities [90]. No *Rhodobacteriaceae* were identified in that study; however, the assembled undermat metagenome showed sixteen annotated rhodopsin genes, affiliated with seven phylogenetic groups (although six rhodopsins were linked to *Armatiomonadetes* members), representing an unexpected diversity of potential retinal-based phototrophy in the mat [90]. These rhodopsin genes were found as part of partial genomes and it was uncertain whether all of these were producing functional rhodopsins. Nevertheless, the unexpected occurrence of rhodopsin- (mainly xanthorhodopsin) type genes in the Mushroom Spring runoff community indicates an important role for rhodopsin-mediated light harvesting in those microbial mats. The samples for the metagenomics study were taken at a higher temperature point (60 °C) than the M37P sampling, which could explain the lack of *Rhodobacter* species in their analysis. 

Although experimental evidence for the exact role of eubacterial rhodopsins is still somewhat scarce, studies with PR from different bacterial organisms have shown an increased survival rate or growth-enhancing effect, particularly in nutrient-poor environments, due to light-induced proton pumping and coupled ATP production via PR [91,92,93,94]. Rhodopsin-containing *Vibrio* species appear to have increased long-term survival under starved conditions when exposed to light, while *Flavobacteria* PR-containing species show a six-fold increase in growth yield under nutrient-poor conditions with light exposure of the appropriate wavelength. Similarly, the presence of XR in *Rba.* M37P is expected to provide either long-term starvation survival or short-term adaptation to low nutrient levels in the Mushroom Spring runoff. Oxygenic cyanobacteria are the predominant primary producers in the Mushroom Spring [90,95]. They are predominantly found in the upper 0–2 mm of the microbial mat where they fix CO_2_ and synthesize and excrete metabolites that are then consumed by (photo)heterotrophic members of the community. This will create a light-dependent diurnal cycling of periods of high and low organic nutrient levels, and rhodopsin-containing species would be expected to have an adaptive advantage if they can extend their growth and energy production under the phases of nutrient-poor conditions. Blue–green light (450–550 nm) is not absorbed as well by the Chl *a*, phycocyanin, and carotenoids of cyanobacteria, and the presence of an antenna chromophore in xanthorhodopsin is certainly expected to be advantageous in niches that receive low irradiance. Since *Rhodobacter* M37P is the only *Rhodobacter* species that contains a xanthorhodopsin gene, it is possible that M37P has selectively obtained this xanthorhodopsin gene cluster through horizontal gene transfer from one of the Mushroom Spring species. In doing so, it thereby obtained a selective advantage in the complex microbial community of the Mushroom Spring runoff area. 

### 3.5. Description of Rhodobacter calidifons sp. nov.

Based on the genomic and genetic differences and the unique growth characteristics described above, it is clear that strain M37P is a distinct new species belonging to the genus *Rhodobacter*. We therefore propose the name *Rhodobacter calidifons* sp. nov. (cali’di.fons; L. masc. adj. *calidi* hot; L. masc. noun *fons* spring; *calidifons* hot spring; named after being isolated from a hot spring runoff). 

Cells are rod-shaped, 1 to 2 μm long, and 0.5 to 0.7 μm wide. Multiplication is by binary fission. Cells appear to be motile. Catalase and oxidase positive. Aerobically grown cultures are white when grown in the light, and pinkish-red pigmented when grown in dark conditions. Colonies are smooth and glossy and dark-pink in dark-incubated cultures. No anaerobic growth in either dark or light conditions. Aerobic anoxygenic phototroph. Optimal pH range, 7.6 to 8.0. Growth temperature range was 30–45 °C, with an optimum temperature of 38–39 °C. Growth occurs on malate, succinate, glutamate, and glucose. Growth factors required are thiamin and nicotinic acid. Yeast extract and biotin stimulate the growth appreciably. Nitrogen sources: ammonium salts and amino acids. Capable of nitrogen fixation and sulfur oxidation. Photopigments: absorption spectra of whole cells show the maxima characteristic of bacteriochlorophyll a (378, 590, 795, and 862 nm). Methanol-extracted pigment from dark-incubated cultures shows absorption maxima at 464, 490, and 524 nm. Contains xanthorhodopsin. DNA base composition: 67.5 mol% G+C (genome analysis) and a genome size of 3.63 Mb.

GenBank accession number of the genome is JAANHS000000000, and GenBank accession number for the full-length 16S rRNA is ON328317. Strain M37P was isolated from runoff from the Mushroom Hot Spring at Yellowstone National Park.

## 4. Conclusions

The lack of anaerobic growth and the effects of light on pigment synthesis and degradation, indicate that *Rhodobacter calidifons* M37P is an aerobic anoxygenic phototroph (AAP). The increased pigment synthesis during dark incubation and decreased pigments observed after several hours of light incubation are consistent with the observed effects in other AAPs and is expected to provide supplemental energy production when light is present in the hot spring runoff. Further physiological studies will be required to clarify the molecular nature of the pigment regulation; however, to our knowledge, this is the only *Rhodobacter* species that has been described as an AAP. Although further characterization of the carotenoids and protein purification will be necessary, based on spectral homology and the presence of a unique xanthorhodopsin-encoding gene, we suggest that the increased pigmentation in *Rba*. *calidifons* M37P is due to the production of xanthorhodopsin. None of the other *Rhodobacter* genomes contain any form of rhodopsin in their genome, nor has any rhodopsin-producing *Rhodobacter* species ever been described. The *Rhodobacter* xanthorodopsin was possibly obtained through horizontal gene transfer from the microbial mat community of the Mushroom Spring runoff, where the presence of a xanthorhodopsin with an antenna chromophore provides a selective advantage under low irradiation. 

AAP bacteria metabolize more efficiently when light is available, suggesting that in oligotrophic waters they can use energy from both light and scarce nutrients simultaneously. In these organisms, light inhibits bacteriochlorophyll a synthesis, so its activity diminishes during the day. On the other hand, rhodopsin-containing cells exploit the light for phototrophy and, due to the light-induced proton pumping, especially in nutrient-poor environments, the cell viability of rhodopsin-containing bacteria increases. The characteristics and adaptation of this unique *Rhodobacter* species indicate that light energy is of major importance in the Mushroom Spring community, even under low light conditions.

## Figures and Tables

**Table 1:** Genomic and growth properties of *Rhodobacter* species. References where the growth data were obtained are included in the table. Other data were obtained from NCBI GenBank and genome analysis performed in PATRIC [32]. LH II = Light Harvesting complex II; Y = positive; N = negative; n.d. = not determined or not available in literature.

Strain	Genome				Growth						Rhodopsin	Rubisco	LH II	Reference
	size (Mb)	GC %	CDS	Genbank Acc.	malate	succinate	glutamate	glucose	temp. optimum	anaerobic growth				
*Rba.* sp. M37P	3.63	67.5	3732	JAANHS000000000	Y	Y	Y	Y	38–39	N	Xantho-rhodopsin	none	N	*this paper*
*Rba. thermarum* YIM73036	3.65	66.8	3695	QMJY00000000	N	Y	N	Y	37–45	N	none	none	N	[9]
*Rba. flagellatus* SYSU G03088	3.62	67.7	3677	VMDU00000000	N	n.d.	N	N	45	n.d.	none	*rbcS*; *rbcL*	N	[16]
*Rba. blasticus* DSM 2131	3.59	66.5	3625	PZKE00000000	Y	n.d.	Y	Y	30–35	Y	none	*rbcII*; *rbcS*; *rbcL*	Y	[8]
*Rba. capsulatus* DSM 1710	3.67	66.5	3601	QKZO00000000	Y	Y	Y	Y	30	Y	none	*rbcII*; *rbcS*; *rbcL*	Y	[1,15]
*Rba. sediminis* SB1003	3.87	66.6	3728	CP001312	Y	n.d.	Y	Y	30–35	Y	none	*rbcII*; *rbcS*; *rbcL*	Y	[20]
*Rba. viridis* JA737	3.87	65.3	3780	QJTK00000000	N	N	N	Y	30	Y	none	*rbcII*	Y	[26]
*Rba. ovatus* JA234	3.81	66.5	3775	OAOQ00000000	N	Y	N	N	25–30	Y	none	*rbcII*; *rbcS*; *rbcL*	Y	[19]
*Rba. vinaykumarii* JA123	3.49	68.2	3366	OBMN00000000	Y	Y	N	Y	20–30	Y	none	*rbcII*; *rbcS*; *rbcL*	Y	[25]
*Rba. maris* JA276	3.83	65.1	3806	OBMT00000000	Y	N	N	N	25–35	Y	none	*rbcII*; *rbcS*; *rbcL*	Y	[18]
*Rba. aestuarii* JA296	3.84	61.1	3742	QAXT00000000	N	N	N	N	25–30	Y	none	*rbcII*; *rbcS*; *rbcL*	Y	[10]
*Rba. sphaeroides* ATH 2.4.1	4.63	68.7	4657	CP030271	Y	Y	Y	Y	30–34	Y	none	*rbcII*; *rbcS*; *rbcL*	Y	[22]
*Rba. sphaeroides* IL106	4.79	69.1	4848	QRBG00000000	Y	Y	Y	Y	30	Y	none	*rbcII*; *rbcS*; *rbcL*	Y	[51]
*Rba. azotoformans* KA25	4.41	68.4	4505	QAOT00000000	Y	Y	Y	Y	30–35	Y	none	*rbcII*; *rbcS*; *rbcL*	Y	[14]
*Rba. johri* JA192	4.59	69.3	4438	PZZW00000000	Y	Y	Y	Y	25–35	Y	none	*rbcII*; *rbcS*; *rbcL*	Y	[17]

## Data Availability

This Whole Genome Shotgun project has been deposited at DDBJ/ENA/GenBank under the accession JAANHS000000000. The version described in this paper is version JAANHS010000000.

## Supplementary Materials

The following supporting information can be downloaded at: https://www.mdpi.com/article/10.3390/microorganisms10061169/s1, Table S1: *Rhodobacter* genomes used for the comparative study. Figure S1: Beta-carotene biosynthesis pathway overview. Enzymes found in the *Rba. calidifons* M37P xanthorhodopsin gene cluster are marked and labeled in pink.

## References

[1] Imhoff J.F., Truper H.G., Pfennig N. (1984). Rearrangement of the species and genera of the phototrophic “Purple Nonsulfur Bacteria”. Int. J. Syst. Bacteriol..

[2] Imhoff J.F., Brenner D.J., Krieg N.R., Staley J.T., Garrity G.M. (2005). Genus *Rhodobacter.* Imhoff, Trüper and Pfennig 1984, 342^VP^. Bergey’s Manual of Systematic Bacteriology.

[3] Kwon S.Y., Jiang S.N., Zheng J.H., Choy H.E., Min J.J. (2014). *Rhodobacter sphaeroides*, a novel tumor-targeting bacteria that emits natural near-infrared fluorescence. Microbiol. Immunol..

[4] Christ W.J., Asano O., Robidoux A.L.C., Perez M., Wang Y., Dubuc G.R., Gavin W.E., Hawkins L.D., McGuinness P.D., Mullarkey M.A. (1995). E5531, a pure endotoxin antagonist of high potency. Science.

[5] A Mullarkey M., Rose J.R., Bristol J.R., Kawata T., Kimura A., Kobayashi S., Przetak M., Chow J., Gusovsky F., Christ W.J. (2003). Inhibition of endotoxin response by e5564, a novel Toll-Like Receptor 4-directed endotoxin entagonist. J. Pharmacol. Exp. Ther..

[6] Kanie Y., Yamaguchi Y., Hayashi A., Uzawa J., Hatakeyama M., Hidaka Y., Toda N., Nakamura S., Kanie O. (2019). Structural analysis of a novel lipooligosaccharide (LOS) from *Rhodobacter azotoformans*. Carbohydr. Res..

[7] Murakami K., Kamimura D., Hasebe R., Uchida M., Abe N., Yamamoto R., Jiang J.J., Hidaka Y., Nakanishi Y., Fujita S. (2021). *Rhodobacter azotoformans* LPS (RAP99-LPS) is a TLR4 agonist that inhibits lung metastasis and enhances TLR3-mediated chemokine expression. Front. Immunol..

[8] Eckersley K., Dow C.S. (1980). *Rhodopseudomonas blastica* sp. nov: A member of the *Rhodospirillaceae*. J. Gen. Microbiol..

[9] Khan I.U., Habib N., Xiao M., Li M.M., Xian W.D., Hejazi M.S., Tarhriz V., Zhi X.Y., Li W.J. (2019). *Rhodobacter thermarum* sp. nov., a novel phototrophic bacterium isolated from sediment of a hot spring. Antonie Van Leeuwenhoek..

[10] Venkata Ramana V., Anil Kumar P., Srinivas T.N., Sasikala C., Ramana C.V. (2009). *Rhodobacter aestuarii* sp. nov., a phototrophic alphaproteo bacterium isolated from an estuarine environment. Int. J. Syst. Evol. Microbiol..

[11] Gandham S., Lodha T., Chintalapati S., Chintalapati V.R. (2018). *Rhodobacter alkalitolerans* sp. nov., isolated from an alkaline brown pond. Arch. Microbiol..

[12] Chen W.M., Chang T.H., Yang C.C., Sheu D.S., Jheng L.C., Sheu S.Y. (2021). *Rhodobacter amnigenus* sp. nov. and *Rhodobacter ruber* sp. nov., isolated from freshwater habitats. Int. J. Syst. Evol. Microbiol..

[13] Suresh G., Sailaja B., Ashif A., Dave B.P., Sasikala C., Ramana C.V. (2017). Description of *Rhodobacter azollae* sp. nov. and *Rhodobacter lacus* sp. nov. Int. J. Syst. Evol. Microbiol..

[14] Hiraishi A., Muramatsu K., Ueda Y. (1996). Molecular genetic analyses of *Rhodobacter azotoformans* sp. nov. and related species of phototrophic bacteria. Syst. Appl. Microbiol..

[15] Molisch H. (1907). Die Purpurbakterien Nach Neuen Untersuchungen.

[16] Xian W.D., Liu Z.T., Li M.M., Liu L., Ming Y.Z., Xiao M., Salam N., Li W.J. (2020). *Rhodobacter flagellatus* sp. nov., a thermophilic bacterium isolated from a hot spring. Int. J. Syst. Evol. Microbiol..

[17] Girija K.R., Sasikala C., Ramana C.V., Spröer C., Takaichi S., Thiel V., Imhoff J.F. (2010). *Rhodobacter johrii* sp. nov., an endospore-producing cryptic species isolated from semi-arid tropical soils. Int. J. Syst. Evol. Microbiol..

[18] Venkata Ramana V., Sasikala C., Ramana C.V. (2008). *Rhodobacter maris* sp. nov., a phototrophic alphaproteobacterium isolated from a marine habitat of India. Int. J. Syst. Evol. Microbiol..

[19] Srinivas T.N., Anil Kumar P., Sasikala C., Spröer C., Ramana C.V. (2008). *Rhodobacter ovatus* sp. nov., a phototrophic alphaproteobacterium isolated from a polluted pond. Int. J. Syst. Evol. Microbiol..

[20] Subhash Y., Lee S.S. (2016). *Rhodobacter sediminis* sp. nov., isolated from lagoon sediments. Int. J. Syst. Evol. Microbiol..

[21] Suresh G., Dhanesh K., Krishnaiah A., Sasikala C., Ramana C.V. (2020). *Rhodobacter sediminicola* sp. nov., isolated from a fresh water pond. Int. J. Syst. Evol. Microbiol..

[22] Van Niel C.B. (1944). The culture, general physiology, morphology and classification of the nonsulfur purple and brown bacteria. Bacteriol. Rev..

[23] Sheu C., Li Z.H., Sheu S.Y., Yang C.C., Chen W.M. (2020). *Tabrizicola oligotrophica* sp. nov. and *Rhodobacter tardus* sp. nov., two new species of bacteria belonging to the family *Rhodobacteraceae*. Int. J. Syst. Evol. Microbiol..

[24] Hansen T.A., Imhoff J.F. (1985). *Rhodobacter veldkampii*, a new species of Phototrophic Purple Nonsulfur Bacteria. Int. J. Syst. Evol. Microbiol..

[25] Srinivas T.N.R., Anil Kumar P., Sasikala C., Ramana C.V., Imhoff J.F. (2007). *Rhodobacter vinaykumarii* sp. nov., a marine phototrophic alphaproteobacterium from tidal waters, and emended description of the genus *Rhodobacter*. Int. J. Syst. Evol. Microbiol..

[26] Raj P.S., Ramaprasad E.V.V., Vaseef S., Sasikala C., Ramana C.V. (2013). *Rhodobacter viridis* sp. nov., a phototrophic bacterium isolated from mud of a stream. Int. J. Syst. Evol. Microbiol..

[27] Han H., Wang T., Chen Z., Li Y., Yao L. (2020). *Rhodobacter xinxiangensis* sp. nov., isolated from pakchoi-cultivated soil contaminated with heavy metal and its potential to reduce Cd and Pb accumulation in pakchoi (*Brassica campestris* L.). Arch. Microbiol..

[28] Suresh G., Lodha T.D., Indu B., Sasikala C., Ramana C.V. (2019). Taxogenomics resolves conflict in the genus *Rhodobacter*: A two and half decades pending thought to reclassify the genus *Rhodobacter*. Front. Microbiol..

[29] Ramaley R.F., Hixson J. (1970). Isolation of a nonpigmented, thermophilic bacterium similar to *Thermus aquaticus*. J. Bacteriol..

[30] Weaver P., Wall J.D., Gest H. (1975). Characterization of *Rhodopseudomonas capsulata*. Arch. Microbiol..

[31] Bankevich A., Nurk S., Antipov D., Gurevich A.A., Dvorkin M., Kulikov A.S., Lesin V.M., Nikolenko S.I., Pham S., Prjibelski A.D. (2012). SPAdes: A new genome assembly algorithm and its applications to single-cell sequencing. J. Comp. Biol..

[32] Wattam A.R., Davis J.J., Assaf R., Boisvert S., Brettin T., Bun C., Conrad N., Dietrich E.M., Disz T., Gabbard J.L. (2017). Improvements to PATRIC, the all-bacterial Bioinformatics Database and Analysis Resource Center. Nucleic Acids Res..

[33] Aziz R.K., Bartels D., Best A.A., DeJongh M., Disz T., Edwards R.A., Formsma K., Gerdes S., Glass E.M., Kubal M. (2008). The RAST server: Rapid annotations using subsystems technology. BMC Genom..

[34] Parks D.H., Imelfort M., Skennerton C.T., Hugenholtz P., Tyson G.W. (2015). CheckM: Assessing the quality of microbial genomes recovered from isolates, single cells, and metagenomes. Genome Res..

[35] Richter M., Rosselló-Móra R., Oliver Glöckner F.O., Peplies J. (2016). JSpeciesWS: A web server for prokaryotic species circumscription based on pairwise genome comparison. Bioinformatics.

[36] Rodriguez-R L.M., Konstantinidis K.T. (2014). Bypassing cultivation to identify bacterial species. Microbe.

[37] Edgar R.C. (2004). MUSCLE: Multiple sequence alignment with high accuracy and high throughput. Nucleic Acids Res..

[38] Stamatakis A., Hoover P., Rougemont J.J.S. (2008). A rapid bootstrap algorithm for the RAxML web servers. Syst. Biol..

[39] Stamatakis A.J.B. (2014). RAxML version 8: A tool for phylogenetic analysis and post-analysis of large phylogenies. Bioinformatics.

[40] Letunic I., Bork P. (2019). Interactive Tree of Life (iTOL) v4: Recent updates and new developments. Nucleic Acids Res..

[41] Thompson J.D., Higgins D.G., Gibson T.J. (1994). CLUSTAL W: Improving the sensitivity of progressive multiple sequence alignment through sequence weighting, position-specific gap penalties and weight matrix choice. Nucleic Acids Res..

[42] Nei M., Kumar S. (2000). Molecular Evolution and Phylogenetics.

[43] Le S.Q., Gascuel O. (2008). An improved general amino acid replacement matrix. Mol. Biol. Evol..

[44] Kumar S., Stecher G., Li M., Knyaz C., Tamura K. (2018). MEGA X: Molecular Evolutionary Genetics Analysis across computing platforms. Mol. Biol. Evol..

[45] Waterhouse A., Bertoni M., Bienert S., Studer G., Tauriello G., Gumienny R., Heer F.T., de Beer T.A.P., Rempfer C., Bordoli L. (2018). SWISS-MODEL: Homology modelling of protein structures and complexes. Nucleic Acids Res..

[46] Camacho C., Coulouris G., Avagyan V., Ma N., Papadopoulos J., Bealer K., Madden T.L. (2009). BLAST+: Architecture and applications. BMC Bioinform..

[47] Steinegger M., Meier M., Mirdita M., Vöhringer H., Haunsberger S.J., Söding J. (2019). HH-suite3 for fast remote homology detection and deep protein annotation. BMC Bioinform..

[48] Mirdita M., von den Driesch L., Galiez C., Martin M.J., Söding J., Steinegger M. (2016). Uniclust databases of clustered and deeply annotated protein sequences and alignments. Nucleic Acids Res..

[49] Studer G., Tauriello G., Bienert S., Biasini M., Johner N., Schwede T. (2021). ProMod3—A versatile homology modelling toolbox. PLoS Comp. Biol..

[50] Studer G., Rempfer C., Waterhouse A.M., Gumienny G., Haas J., Schwede T. (2020). QMEANDisCo—Distance constraints applied on model quality estimation. Bioinformatics.

[51] Satoh T., Hoshino Y., Kitamura H. (1976). *Rhodopseudomonas sphaeroides* forma sp. denitrificans, a denitrifying strain as a subspecies of *Rhodopseudomonas sphaeroides*. Arch. Microbiol..

[52] Stackebrandt E., Ebers J. (2006). Taxonomic parameters revisited: Tarnished gold standards. Microbiol. Today.

[53] Yurkov V.V., Beatty J.T. (1998). Aerobic Anoxygenic Phototropic Bacteria. Microbiol. Mol. Biol. Rev..

[54] Yurkov V., Hughes E. (2013). Genes associated with the peculiar phenotypes of the Aerobic Anoxygenic Phototrophs. Advances in Botanical Research.

[55] Kusian B., Bowien B. (1997). Organization and regulation of CBB CO_2_ assimilation genes in autotrophic bacteria. FEMS Microbiol. Rev..

[56] Ashida H., Danchin A., Yokota A. (2005). Was photosynthetic RuBisCO recruited by acquisitive evolution from RuBisCO-like proteins involved in sulfur metabolism?. Res. Microbiol..

[57] Badger M.R., Bek E.J. (2008). Multiple Rubisco forms in proteobacteria: Their functional significance in relation to CO_2_ acquisition by the CBB cycle. J. Exp. Bot..

[58] Kyndt J.A., Aviles F.A., Imhoff J.F., Künzel S., Neulinger S.C., Meyer T.E. (2022). Comparative genome analysis of the photosynthetic betaproteobacteria of the genus *Rhodocyclus*: Heterogeneity within strains assigned to *Rhodocyclus tenuis* and description of *Rhodocyclus gracilis* sp. nov. as a new species. Microorganisms.

[59] Imker H.J., Fedorov A.A., Fedorov E.V., Almo S.C., Gerlt J.A. (2007). Mechanistic diversity in the RuBisCO superfamily: The “enolase” in the methionine salvage pathway in *Geobacillus kaustophilus*. Biochemistry.

[60] Pemberton J.M., Horne I.M., McEwan A.G. (1998). Regulation of photosynthetic gene expression in purple bacteria. Microbiology.

[61] Gregor J., Klug G. (1999). Regulation of bacterial photosynthesis genes by oxygen and light. FEMS Microbiol. Lett..

[62] Yurkov V., van Gemerden H. (1993). Impact of light/dark regime on growth rate, biomass formation and bacteriochlorophyll synthesis in *Erythromicrobium hydrolyticum*. Arch. Microbiol..

[63] Takamiya K.I., Shioi Y., Shimada H., Arata H. (1992). Inhibition of accumulation of bacteriochlorophyll and carotenoids by blue light in an aerobic photosynthetic bacterium *Roseobacter denitrificans*, during anaerobic respiration. Plant Cell Physiol..

[64] Yurkov V., Krasilnikova E.N., Gorlenko V.M. (1993). Effect of light and oxygen on metabolism of the aerobic bacterium *Erythromicrobium sibiricum*. Microbiology.

[65] Cartron M.L., Olsen J.D., Sener M., Jackson P.J., Brindley A.A., Qian P., Dickman M.J., Leggett G.J., Schulten K., Neil Hunter C. (2014). Integration of energy and electron transfer processes in the photosynthetic membrane of *Rhodobacter sphaeroides*. Biochim. Biophys. Acta Bioenerg..

[66] Olsen J.D., Tucker J.D., Timney J.A., Qian P., Vassilev C., Hunter C.N. (2008). The organization of LH2 complexes in membranes from *Rhodobacter sphaeroides*. J. Biol. Chem..

[67] Gabrielsen M., Gardiner A.T., Cogdell R., Hunter C.N., Daldal F., Thurnauer M.C., Beatty J.T. (2009). Peripheral complexes of purple bacteria. The Purple Phototrophic Bacteria, Advances in Photosynthesis and Respiration.

[68] Jaschke P.R., Leblanc H.N., Lang A.S., Beatty J.T. (2008). The PucC protein of *Rhodobacter capsulatus* mitigates an inhibitory effect of light-harvesting 2 alpha and beta proteins on light-harvesting complex 1. Photosynth Res..

[69] Mothersole D.J., Jackson P.J., Vasilev C., Tucker J.D., Brindley A.A., Dickman M.J., Hunter C.N. (2016). PucC and LhaA direct efficient assembly of the light-harvesting complexes in *Rhodobacter sphaeroides*. Mol. Microbiol..

[70] Selyanin V., Hauruseu D., Koblížek M. (2016). The variability of light-harvesting complexes in aerobic anoxygenic phototrophs. Photosynth Res..

[71] Wakao N., Shiba T., Hiraishi A., Ito M., Sakurai Y. (1993). Distribution of bacteriochlorophyll a in species of the genus *Acidiphilium*. Curr. Microbiol..

[72] Wakao N., Yokoi N., Isoyama N., Hiraishi A., Shimada K., Kobayashi M., Kise H., Iwaki M., Itoh S., Takaichi S. (1996). Discovery of natural photosynthesis using Zn-containing bacteriochlorophyll in an aerobic bacterium *Acidiphilium rubrum*. Plant Cell Physiol..

[73] Koblížek M. (2015). Ecology of aerobic anoxygenic phototrophs in aquatic environments. FEMS Microbiol. Rev..

[74] Bekker A., Holland H.D., Wang P.L., Rumble D., Stein H.J., Hannah J.L., Coetzee L.L., Beukes N.J. (2004). Dating the rise of atmospheric oxygen. Nature.

[75] Koblížek M., Zeng Y., Horák A., Oborník M., Thomas Beatty J. (2013). Regressive evolution of photosynthesis in the *Roseobacter* clade. Advances in Botanical Research.

[76] Balashov S.P., Imasheva E.S., Boichenko V.A., Antón J., Wang J.M., Lanyi J.K. (2005). Xanthorhodopsin: A proton pump with a light-harvesting carotenoid antenna. Science.

[77] Lanyi J.K., Balashov S.P. (2008). Xanthorhodopsin: A bacteriorhodopsin-like proton pump with a carotenoid antenna. Biochim. Biophys. Acta.

[78] Morizumi T., Ou W.L., Van Eps N., Inoue K., Kandori H., Brown L.S., Ernst O.P. (2019). X-ray crystallographic structure and oligomerization of *Gloeobacter* rhodopsin. Sci. Rep..

[79] Zhong Z.-P., Liu Y., Wang F., Zhou Y.-G., Liu H.-C., Liu Z.-P. (2016). *Aquisalinus flavus* gen. nov., sp. nov., a member of the family *Parvularculaceae* isolated from a saline lake. Int. J. Syst. Evol. Microbiol..

[80] Liu Z.X., Dorji P., Liu H.C., Li A.H., Zhou Y.G. (2019). *Tabrizicola sediminis* sp. nov., one aerobic anoxygenic photoheterotrophic bacteria from sediment of saline lake. Int. J. Syst. Evol. Microbiol..

[81] Woggon W.-D. (2002). Oxidative cleavage of carotenoids catalyzed by enzyme models and beta-carotene 15,15′-monooxygenase. Pure Appl. Chem..

[82] Kim J., Smith J.J., Tian L., Dellapenna D. (2009). The evolution and function of carotenoid hydroxylases in *Arabidopsis*. Plant Cell Physiol..

[83] Canniffe D.P., Hitchcock A., Jez J. (2021). Photosynthesis: Carotenoids in Photosynthesis—Structure and Biosynthesis. Encyclopedia of Biological Chemistry III.

[84] Maresca J.A., Graham J.E., Wu M., Eisen J.A., Bryant D.A. (2007). Identification of a fourth family of lycopene cyclases in photosynthetic bacteria. Proc. Natl. Acad. Sci. USA.

[85] Frigaard N.U., Martinez A., Mincer T.J., DeLong E.F. (2006). Proteorhodopsin lateral gene transfer between marine planktonic Bacteria and Archaea. Nature.

[86] Finkel O., Béjà O., Belkin S. (2013). Global abundance of microbial rhodopsins. ISME J..

[87] Gushchin I., Shevchenko V., Polovinkin V., Kovalev K., Alekseev A., Round E., Borshchevskiy V., Balandin T., Popov A., Gensch T. (2015). Crystal structure of a light-driven sodium pump. Nat. Struct. Mol. Biol..

[88] Kato H.E., Inoue K., Abe-Yoshizumi R., Kato Y., Ono H., Konno M., Hososhima S., Ishizuka T., Hoque M.R., Kunitomo H. (2015). Structural basis for Na(+) transport mechanism by a light-driven Na(+) pump. Nature.

[89] Boichenko V.A., Wang J.M., Antón J., Lanyi J.K., Balashov S.P. (2006). Functions of carotenoids in xanthorhodopsin and archaerhodopsin, from action spectra of photoinhibition of cell respiration. Biochim. Biophys. Acta–Bioenerg..

[90] Thiel V., Hügler M., Ward D.M., Bryant D.A. (2017). The dark side of the Mushroom Spring microbial mat: Life in the shadow of Chlorophototrophs. II. Metabolic functions of abundant community members predicted from metagenomic snalyses. Front. Microbiol..

[91] Gómez-Consarnau L., González J.M., Coll-Lladó M., Gourdon P., Pascher T., Neutze R., Pedrós-Alió C., Pinhassi J. (2007). Light stimulates growth of proteorhodopsin-containing marine *Flavobacteria*. Nature.

[92] Gómez-Consarnau L., Akram N., Lindell K., Pedersen A., Neutze R., Milton D.L., González J.M., Pinhassi J. (2010). Proteorhodopsin phototrophy promotes survival of marine bacteria during starvation. PLoS Biol..

[93] Wang Z., O’Shaughnessy T.J., Soto C.M., Rahbar A.M., Robertson K.L., Lebedev N., Vora G.J. (2012). Function and regulation of *Vibrio campbellii* Proteorhodopsin: Acquired phototrophy in a classical organoheterotroph. PLoS ONE.

[94] Kumagai Y., Yoshizawa S., Nakajima Y., Watanabe M., Fukunaga T., Ogura Y., Hayashi T., Oshima K., Hattori M., Ikeuchi M. (2018). Solar-panel and parasol strategies shape the proteorhodopsin distribution pattern in marine *Flavobacteria*. ISME J..

[95] Thiel V., Wood J.M., Olsen M.T., Tank M., Klatt C.G., Ward D.M., Bryant D.A. (2016). The dark side of the Mushroom Spring microbial mat: Life in the shadow of Chlorophototrophs. I. Microbial diversity based on 16S rRNA gene amplicons and metagenomic sequencing. Front. Microbiol..

