# Supplementary material for: Genome Sequence and Characterization of a Xanthorhodopsin-Containing, Aerobic Anoxygenic Phototrophic Rhodobacter Species, Isolated from Mesophilic Conditions at Yellowstone National Park
Source: Microorganisms. 2022 Jun 7;10(6):1169. doi: 10.3390/microorganisms10061169 (PMC9231093; doi:10.3390/microorganisms10061169)
Supplement: Supplementary file 1 [file microorganisms-10-01169-s001.zip › Supplementary Table S1.pdf]

Table S1. *Rhodobacter* genomes used for the comparative study.

Genome ID	Genome Name	Strain	NCBI Taxon ID	Genome Status
1054202.4	Rhodobacter viridis strain JA737	JA737	1054202	WGS
1061.5	Rhodobacter capsulatus strain B41	B41	1061	WGS
1061.6	Rhodobacter capsulatus strain A52	A52	1061	WGS
1061.9	Rhodobacter capsulatus strain DSM 1710	DSM 1710	1061	WGS
1062.4	Rhodobacter sp. strain UBA9917	UBA9917	1062	WGS
1062.5	Rhodobacter sp. strain UBA12248	UBA12248	1062	WGS
1062.6	Rhodobacter sp. strain UBA9318	UBA9318	1062	WGS
1062.8	Rhodobacter sp. strain UBA10365	UBA10365	1062	WGS
1063.1	Rhodobacter sphaeroides strain MBTLJ-13	MBTLJ-13	1063	Complete
1063.13	Rhodobacter sphaeroides strain MBTLJ-20	MBTLJ-20	1063	Complete
1063.28	Rhodobacter sphaeroides strain YL101	YL101	1063	WGS
1063.31	Rhodobacter sphaeroides strain org#2181 strain 28/5	28/5	1063	Complete
1063.32	Rhodobacter sphaeroides strain S2_003_000_R2_11	S2_003_000_	1063	WGS
1063.33	Rhodobacter sphaeroides strain EBL0706	EBL0706	1063	Complete
1063.34	Rhodobacter sphaeroides strain FY	FY	1063	WGS
1063.35	Rhodobacter sphaeroides strain SCJ	SCJ	1063	WGS
1063.4	Rhodobacter sphaeroides strain AB25	AB25	1063	Complete
1063.41	Rhodobacter sphaeroides strain AB27	AB27	1063	Complete
1063.42	Rhodobacter sphaeroides strain AB29	AB29	1063	Complete
1063.43	Rhodobacter sphaeroides strain AB24	AB24	1063	Complete
1063.9	Rhodobacter sphaeroides strain MBTLJ-8	MBTLJ-8	1063	Complete
1185920.4	Rhodobacter veldkampii DSM 11550	DSM 11550	1185920	WGS
1188249.3	Cereibacter changlensis JA139	JA139	1188249	WGS
1188250.3	Rhodobacter blasticus DSM 2131	DSM 2131	1188250	WGS
1247725.3	Rhodobacter sp. AKP1	AKP1	1247725	WGS
1414586.3	Rhodobacter capsulatus YW1	YW1	1414586	WGS
1415159.3	Rhodobacter capsulatus YW2	YW2	1415159	WGS
1415160.3	Rhodobacter capsulatus DE442	DE442	1415160	WGS
1415161.3	Rhodobacter capsulatus Y262	Y262	1415161	WGS
1415162.4	Rhodobacter capsulatus B6	B6	1415162	WGS
1415163.3	Rhodobacter capsulatus R121	R121	1415163	WGS
1655557.3	Rhodobacter sp. BACL10 MAG-120419-bin15	BACL10 MAG	1655557	WGS
1786003.4	Rhodobacter sp. CCB-MM2	CCB-MM2	1786003	WGS
1850250.3	Rhodobacter sp. LPB0142	LPB0142	1850250	Complete
1884310.3	Rhodobacter sp. 24-YEA-8	24-YEA-8	1884310	WGS
1985673.3	Rhodobacter sp. CCP-1	CCP-1	1985673	WGS
2029399.6	Rhodobacter sp. TJ_12	TJ_12	2029399	WGS
2033869.3	Rhodobacter sp. CZR27	CZR27	2033869	Complete
2183910.3	Rhodobacter sp. 140A	140A	2183910	WGS
2249421.3	Rhodobacter sp. YIM 73036	YIM 73036	2670345	WGS
2294033.3	Rhodobacter sp. BO-81	BO-81	2294033	WGS
2528036.3	Rhodobacter sp. YIM 73028	YIM 73028	2528036	WGS
2562317.3	Rhodobacter sp. SY28-1	SY28-1	2562317	WGS
2584941.3	Rhodobacter sp. JA983	JA983	2584941	WGS
2593019.3	Rhodobacter sp. SYSU G02092	SYSU G0209	2593019	WGS
2593020.3	Rhodobacter sp. SYSU G02094	SYSU G0209	2593020	WGS
2593021.3	Rhodobacter sp. SYSU G03088	SYSU G0308	2593021	WGS
2593022.3	Rhodobacter sp. SYSU G02214	SYSU G0221	2593022	WGS
2715277.3	Rhodobacter sp. M37P	M37P	2715277	WGS
272942.6	Rhodobacter capsulatus SB 1003	SB1003	272942	Complete
272943.71	Rhodobacter sphaeroides 2.4.1	2.4.1	272943	Complete
2759676.3	Rhodobacter sp. NTK016B	NTK016B	2759676	WGS
2807096.3	Rhodobacter sp. N10	N10	2807096	Complete
2852097.3	Rhodobacter sp. HSP-20	HSP-20	2852097	WGS
2883999.5	Rhodobacter sp. Har01	Har01	2883999	WGS
349101.6	Rhodobacter sphaeroides ATCC 17029	ATCC 17029	349101	Complete
349102.13	Rhodobacter sphaeroides ATCC 17025	ATCC 17025	349102	Complete
371731.3	Rhodobacter sp. SW2	SW2	371731	WGS
39723.4	Rhodobacter sphaeroides f. sp. denitrificans strain IL106	IL106	39723	WGS
407234.3	Rhodobacter vinaykumarii strain DSM 18714	DSM 18714	407234	WGS
407234.6	Rhodobacter vinaykumarii strain JA123	JA123	407234	WGS
418630.3	Rhodobacter megalophilus strain DSM 18937	DSM 18937	418630	WGS
43057.4	Rhodobacter azotoformans strain YLK20	YLK20	43057	WGS
43057.6	Rhodobacter azotoformans strain KA25	KA25	43057	WGS
439529.3	Rhodobacter ovatus strain JA234	JA234	439529	WGS
445629.8	Rhodobacter johrii strain JA192	JA192	445629	WGS
445629.9	Rhodobacter johrii strain CDR-SL 7Cii	CDR-SL 7Cii	445629	WGS
446682.3	Rhodobacter maris strain JA276	JA276	446682	WGS
449393.3	Rhodobacter sp. Q27b	Q27b	1408890	WGS
453582.3	Rhodobacter aestuarii strain DSM 19945	DSM 19945	453582	WGS
453582.5	Rhodobacter aestuarii strain JA296	JA296	453582	WGS
557760.5	Rhodobacter sphaeroides KD131	KD131	557760	Complete
570013.3	Rhodobacter sp. JA431	JA431	570013	WGS
992186.3	Rhodobacter sphaeroides WS8N	WS8N	992186	WGS
