# Supplementary figures and images for: Genome Sequence and Characterization of a Xanthorhodopsin-Containing, Aerobic Anoxygenic Phototrophic Rhodobacter Species, Isolated from Mesophilic Conditions at Yellowstone National Park
Source: Microorganisms. 2022 Jun 7;10(6):1169. doi: 10.3390/microorganisms10061169 (PMC9231093; doi:10.3390/microorganisms10061169)

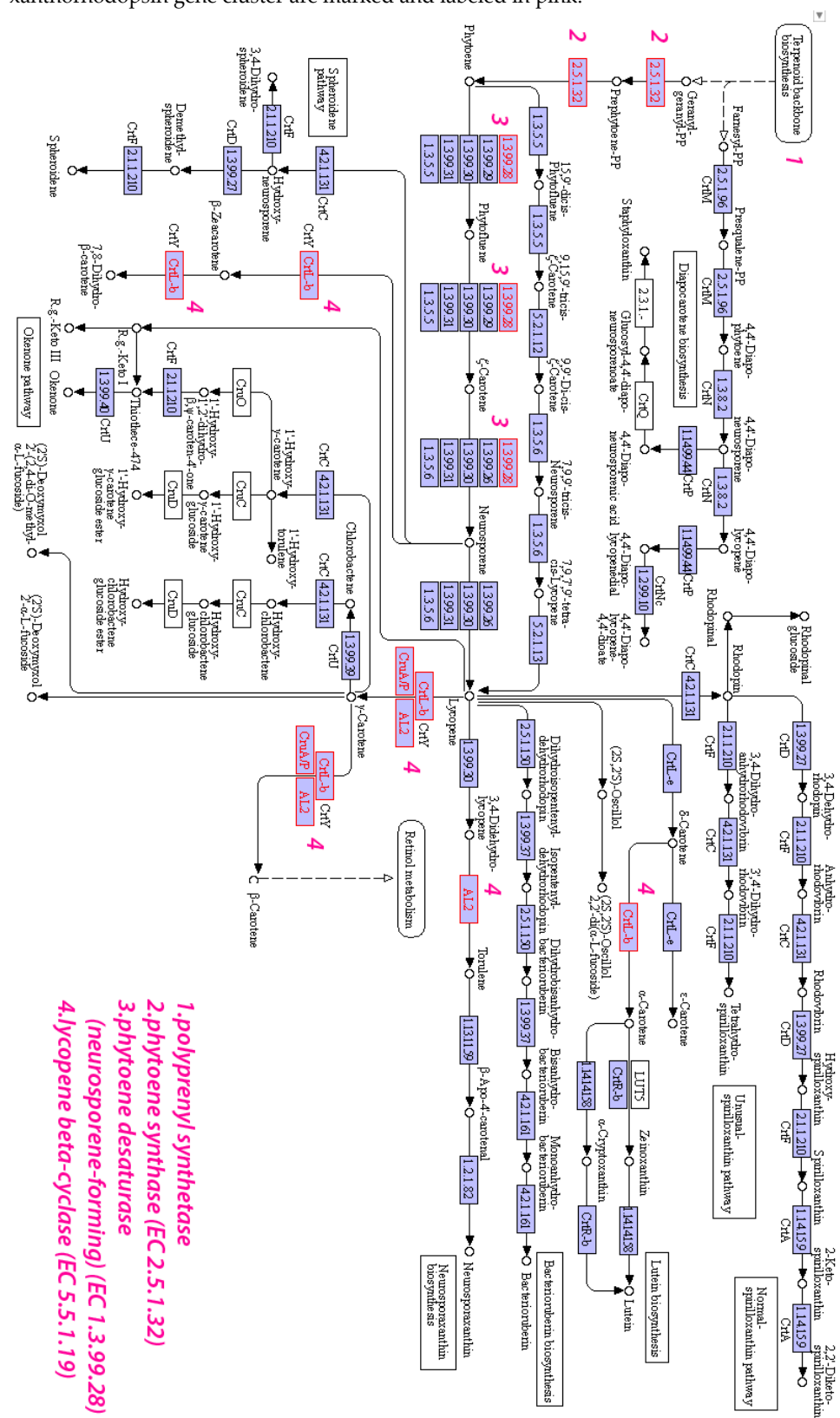

Supplement: Supplementary file 1 [file microorganisms-10-01169-s001.zip › Supplementary Figure S1.pdf]
